# Pleasurable music activates cerebral µ-opioid receptors: a combined PET-fMRI study

**DOI:** 10.1007/s00259-025-07232-z

**Published:** 2025-04-04

**Authors:** Vesa Putkinen, Kerttu Seppälä, Harri Harju, Jussi Hirvonen, Henry K. Karlsson, Lauri Nummenmaa

**Affiliations:** 1 Turku PET Centre, and Turku University Hospital, University of Turku, Turku, Finland; 2 Turku Institute for Advanced Studies, University of Turku, Turku, Finland; 3 Department of Medical Physics, Turku University Hospital, Turku, Finland; 4 Department of Radiology, Turku University Hospital, University of Turku, Turku, Finland; 5 Medical Imaging Center, Department of Radiology, Tampere University and Tampere University Hospital, Tampere, Finland; 6 Department of Psychology, University of Turku, Turku, Finland; 7 Department of Adolescent Psychiatry, University of Turku, Turku, Finland; 8 Wellbeing Services County of Satakunta, Psychiatric Care Division, Pori, Finland

**Keywords:** Music, Pleasure, Endogenous Opioids, MOR, PET, fMRI

## Abstract

**Purpose:**

The μ-opioid receptor (MOR) system mediates incentive motivation and the hedonic component of primary rewards such as food and sex. However, there is no direct in vivo evidence for the involvement of the MOR system in pleasure derived from aesthetic rewards such as music.

**Methods:**

We measured MOR availability with positron emission tomography (PET) and the agonist radioligand [^11^C]carfentanil with high affinity for MORs during the listening of pleasurable music and neutral baseline condition. Haemodynamic responses associated with dynamic pleasure ratings during listening to music and control stimuli were measured using functional magnetic resonance imaging (fMRI).

**Results:**

The PET results revealed that pleasurable music increased [^11^C]carfentanil binding in several cortical and subcortical regions, including ventral striatum and orbitofrontal cortex, known to contain “hedonic hotspots”. [^11^C]carfentanil binding in the nucleus accumbens during the music condition was associated with number of pleasurable chills, linking the subjective experience of pleasure to striatal opioid release. Individual variation in baseline MOR tone influenced pleasure-dependent haemodynamic responses during music listening in regions associated with interoceptive, sensorimotor, and reward processing.

**Conclusions:**

These findings provide the first neuroimaging evidence that pleasurable music modulates MOR system function. The results indicate that the μ-opioid system governs complex aesthetic rewards in addition to biologically essential primary rewards.

**Supplementary Information:**

The online version contains supplementary material available at 10.1007/s00259-025-07232-z.

## Introduction

Pleasure motivates humans to seek out rewards crucial for survival and reproduction. Yet, human hedonic responses extend beyond such primary rewards and encompass abstract, aesthetic rewards that seemingly lack adaptive function. A prime example is music, which has persisted throughout human evolution and remains one of the most common sources of pleasure today despite offering no obvious survival advantage (however, see refs [1, 2]). Nevertheless, neuroimaging studies indicate that music-induced pleasure engages the same hedonic circuitry as biologically salient primary rewards, including regions such as the ventral striatum, orbitofrontal cortex, and insula [3–5]. However, the majority of these studies have been conducted using functional magnetic resonance imaging (fMRI), which is unable to resolve the underlying neurochemistry (however, see [6]). Given the importance of specific neuromodulator systems in governing human emotion [7, 8], unraveling the molecular neural basis of musical hedonia is crucial for understanding the universal human affinity towards music.

The μ-opioid receptor (MOR) system plays a central role in incentive motivation and the hedonic components of primary rewards. Studies in rodents indicate that subregions in the nucleus accumbens, ventral pallidum, insula, and orbitofrontal cortex contain cell assemblies whose activity is causally related to hedonic reactions [9]. Injection of opioids into these regions heightens liking behaviors, indicating that MORs mediate pleasure associated with reward consumption [10]. In humans, opioid agonists and antagonists modulate the hedonic impact of rewards like sweet taste [11] and sexual stimuli [12]. Multivariate pattern analysis of fMRI data has revealed that activity associated with subjective pleasure overlaps with regions showing mu-opioid receptor gene expression and is down-regulated by an opioid antagonist [13]. Positron emission tomography (PET) studies using the MOR-specific radioligand [^11^C]carfentanil have revealed opioid release following biologically salient rewards such as eating and sex [14, 15], and baseline MOR availability modulates heamodynamic and behavioral responses to emotional stimuli [15, 16]. Although the contribution of the MOR system musical hedonia has also been postulated [5, 17], this hypothesis remains to be tested with in vivo neuroimaging.

Several lines of evidence suggest, however, that the MOR system might be engaged during musical hedonia. First, given that the MOR system mediates analgesia, the pain-relieving and prosocial effects of pleasurable music lend indirect support for opioidergic contribution to music-induced pleasure [5, 17]: Listening to pleasurable music alleviates chronic and post-operative pain and reduces the need for opioid pain medication in patients [18, 19] while engaging in musical activities such as singing and dancing heightens pain threshold in healthy populations [20]. Secondly, musical activities foster social bonding and promote cooperation [20–22], and the involvement of opioids in affiliative behavior, established through pharmacological interventions in non-human primates [23, 24] and PET studies in humans [15, 25], suggests that the MOR systems may mediate these prosocial effects of music. Finally, pharmacological down- and up-regulation of endogenous opioid function have yielded partial support for opioidergic contribution to music-induced pleasure. The earliest study [26] observed diminished music-induced pleasure after opioid receptor blockade with naltrexone. Two subsequent pharmacological studies, however, failed to replicate the effect on subjective pleasure but did observe that opioid agonists and/or antagonists modulated neurophysiological markers of music-induced arousal [27, 28].

### The current study

To directly test whether musical pleasures engage the MOR system, we measured MOR availability in vivo with PET using the high-affinity agonist radioligand [^11^C]carfentanil while participants listened to self-selected pleasurable music versus a neutral control condition. We also measured haemodynamic responses to the same musical excerpts in a separate fMRI experiment to unravel the interplay between the MOR system and the acute pleasure-dependent haemodynamic activity. We also quantified autonomic nervous system activity during music listening by measuring heart rate and pupil size. Pleasurable music increased MOR availability compared to a neutral baseline condition, particularly in brain regions linked to emotion and reward, such as the ventral striatum, amygdala, and orbitofrontal cortex. We hypothesized that subjective experiences of pleasurable music-induced chills would be associated with [^11^C]carfentanil binding in the nucleus accumbens. Consistent with this hypothesis, subjective experience of music-induced chills during the music scan was negatively associated with [^11^C]carfentanil binding in the nucleus accumbens. Pleasure-dependent hemodynamic responses were observed in somatosensory and emotion-related networks, with greater intensity in participants with higher baseline MOR availability. The heart rate and pupil size measurements indicated that pleasurable music induced heightened autonomic arousal. These data indicate that the MOR system significantly contributes to musical pleasure and that individual variation in MOR availability may constitute a molecular mechanism explaining individual differences in the propensity for enjoying music.

## Methods

Details of subject recruitment, PET image processing, MRI data acquisition and preprocessing, and heart rate and pupil size measurements can be found in the Supplementary Methods.

### Participants

Fifteen women (mean age 26.0 years, range 19–42) participated in both the PET and fMRI experiments. Fifteen additional women (mean age 23.33 years, range 19–27) participated in the fMRI. All the fMRI-only participants as along with eighteen additional women who were not involved in either brain imaging experiment (mean age 25.7 years, range 20–37), took part in the eye-tracking experiment (see Supplementary Methods for more details). All participants gave informed, written consent and were compensated for their participation. The ethics board of the Hospital District of Southwest Finland had approved the protocol, and the study was conducted in accordance with the Declaration of Helsinki.

### Stimuli

We used self-selected music as stimuli to maximize their emotional impact [6]. Each subject compiled an approximately 90-min playlist of music that elicited strong pleasure in them. Most stimuli were contemporary pop, R&B, and rap/hip-hop (See Figure S1).

### PET experimental protocol

Participants took part in two PET 51-min scans: a music (“challenge”) scan and a neutral baseline scan. Before the music scan, the subject listened to their playlist through headphones alone for 15 min. During the PET acquisition, the participants lay in the PET scanner wearing hospital clothes and continued to listen to their playlist through headphones. The baseline scan was otherwise identical but without the musical stimulation. During both scans, the participants were asked to verbally report their level of felt pleasure on a scale of 1–10 (no pleasure to high pleasure) every 10 min. The participants also reported the occurrence of music-induced chills with a button box. The pleasure ratings during the baseline scan were intended to capture participants' subjective experiences of pleasure that occurred in the absence of music stimulation, ensuring consistency in data collection across the scans, and allowing us to compare subjective pleasure levels between baseline and music conditions. The music and baseline scans were completed on separate days at the same time of the day, and the order of the scans was counterbalanced across participants.

### PET data acquisition

MOR availability was measured with radioligand [^11^C]carfentanil synthesized as described previously [29]. The radiochemical purity of the produced [^11^C]carfentanil batches was 98.1 ± 0.4% (mean ± SD). The injected [^11^C]carfentanil radioactivity was 252 ± 10 MBq, and molar radioactivity at the time of injection was 354 ± 240 MBq/nmol, corresponding to an injected mass of 0.52 ± 0.43 µg. PET imaging was carried out with Discovery 690 PET/CT scanner (GE Healthcare, US). The tracer was administered as a single bolus via a catheter placed in the participant’s antecubital vein, and radioactivity was monitored for 51 min. The participant’s head was strapped to the scan table to prevent excessive head movement. T1-weighted MR scans were obtained for attenuation correction and anatomical reference.

### Statistical analysis of the PET data

The voxel-vise differences between the music and baseline conditions in MOR availability were assessed in SPM12 (http://www.fil.ion.ucl.ac.uk/spm/) using a repeated-measures t-test. The statistical threshold was set at *p* < 0.05, FWE-corrected at the cluster level. We correlated the number of chills experienced and the mean *BP*_ND_ during the music condition in left and right nucleus accumbens (NAcc) with masks from the Harvard–Oxford anatomical atlas. Chills have been widely used as a measure of intense music-induced pleasure, and the NAcc was selected as a region-of-interest (ROI) due to strong evidence linking it to both music-induced chills and pleasure-related opioid release [6, 9]. We also ran an exploratory whole-brain voxel-wise second-level analysis on the *BP*_ND_ maps obtained in the music condition using the number of chills as a between subjects-covariate.

### fMRI experimental protocol

The 15-min fMRI experiment was run on a separate day after the PET scans. The participants were presented with ten 45-s segments extracted from their self-selected pleasurable musical pieces (see above). They were also presented with two 45-s random tone sequences as control stimuli. Tones composing the sequences included the fundamental and two harmonics and were 0.1–1 s in duration and 110 Hz to 1864 Hz in pitch, corresponding to musical notes of A2 to A#6. Stimuli were presented binaurally via MRI-compatible headphones (Sensimetrics S14) at a comfortable level adjusted individually for each participant. Participants were asked to remain still during the scan and focus on the feelings evoked by the music. The subject used a button box to move a cursor on the screen to indicate the current music-induced pleasure from 0 (no pleasure) to 10 (high pleasure) during the music and the control stimulation blocks. The starting position of the cursor was randomized. The stimulation blocks were presented in a random order and were interspersed with 30 s rest blocks with no stimulation.

### Regional effects in the general linear model

The fMRI data were analyzed in SPM12 (Wellcome Trust Center for Imaging, London, UK, (http://www.fil.ion.ucl.ac.uk/spm). To reveal regions whose activity varied with music-induced pleasure across the music and control blocks, a general linear model (GLM) was fit to the data where the design matrix, including a boxcar regressor for the music block vs. silent periods with subjective pleasure rating across the whole scan as a parametric modulator of interest (cf. [30]). Note that these dynamic pleasure ratings were used as regressors of interest not only in the music blocks but also in the control (silent) blocks, enabling us to localize regions whose activity increased with increasing pleasure across both conditions. A stick function nuisance regressor for the button presses was also included. For each subject, contrast images were generated for the main effect of pleasure. The contrast images were then subjected to a second-level analysis for population-level inference. Clusters surviving family-wise error rate (FWE) correction (*p* < 0.05) are reported.

### PET-MRI fusion analysis

To examine the connection between baseline MOR tone and hemodynamic responses in music-induced pleasure, we calculated mean subject-wise baseline MOR availabilities across the 17 ROIs (amygdala, caudate, cerebellum, dorsal anterior cingulate cortex, inferior temporal cortex, insula, middle temporal cortex, nucleus accumbens, orbitofrontal cortex, pars opercularis, posterior cingulate cortex, putamen, rostral anterior cingulate cortex, superior frontal gyrus, superior temporal sulcus, temporal pole, and thalamus) defined by the FreeSurfer parcellations. As in previous studies (e.g. ref [31]), baseline MOR availability was used, as it provides a measure of an individual's trait-like MOR tone unlike MOR availability during the challenge, which also reflects music-induced changes in *BP*_ND._ We then correlated the subject-wise MOR availabilities in all ROIs with the voxel-wise pleasure-dependent fMRI beta maps, allowing us to identify regions where the BOLD response was significantly associated with MOR availability in each ROI. Finally, we thresholded and binarized these ROI-wise MOR-dependent BOLD response maps and generated a cumulative map to illustrate the brain regions where haemodynamic activity was consistently associated with MOR availability across ROIs.

## Results

### Subjective pleasure evoked by music

**Fig. 1:**
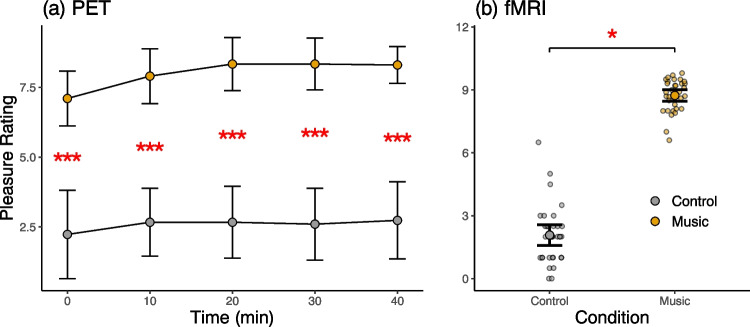
Pleasure ratings during the PET scans for the music and control conditions (**a**) and the music and control blocks in fMRI (**b**). The error bars show 95% confidence intervals. *** = *p* < 0.001, * = *p* < 0.05

We analyzed pleasure ratings obtained during the PET scans with a linear mixed-effect model with Time (0, 10, 20, 30, 40 min) and Condition (music vs. baseline) as fixed factors and subject as a random factor (pleasure ~ time * condition + (1|subject)). Participants experienced significantly more pleasure during the music scan than during the baseline scan (Main effects of Condition: F(1, 32) = 45.135, *p* < 0.001, Fig. 1a). The participants reported an average of 6 (SD = 4.80) chills during the music scan. We extracted the maximum pleasure rating for the music and control blocks from the continuous ratings obtained during the fMRI scan and then calculated separate averages for the music and control conditions for each subject. A repeated measures t-test indicated that the ratings were higher for the music than for the control condition (t(32) = 28.504, *p* < 0.001) (Fig. 1b).

### Eye tracking and heart rate

**Fig. 2:**
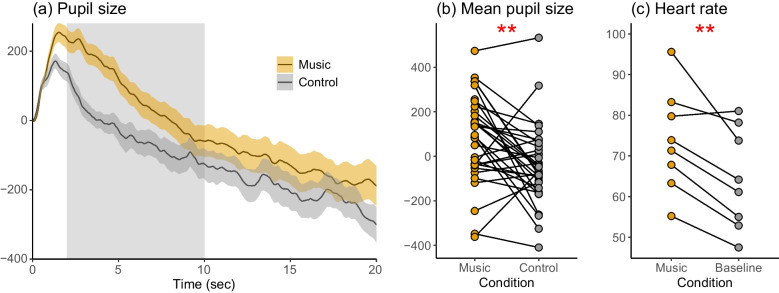
(**a**) Average pupil size as a function of time for the pleasurable music excerpts and neutral control stimuli during the eye-tracking experiment. The shaded area around the curves indicates 95% confidence interval. The shaded rectangle indicates the time window (2–10 s) used to quantify mean pupil size (**b**) The mean pupil size per subject for the music and control stimuli (**c**) The mean heart rate during the Music and Baseline PET scans. ** = *p* < 0.01

In the eye-tracking experiment, the participants’ pupil size was significantly larger for the music excerpts than for the control stimuli between 2–10 s from stimulus onset (*t*(32) = 3.268, *p* < 0.01.) (Fig. 2a and b). During the PET scans, the participants’ mean heart rate was higher for the Music than for the Baseline scans (*t*(7) = 4.104, *p* < 0.01.) (Fig. 2c).

### PET

**Fig. 3:**
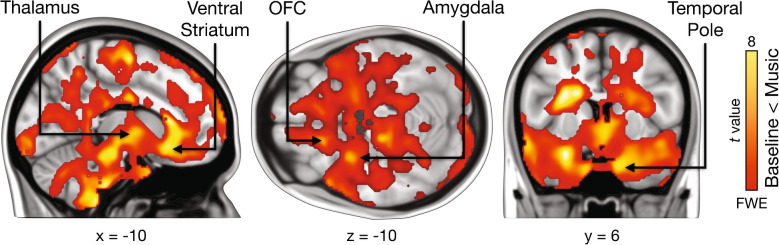
Regions showing increased *BP*_ND_ during music listening relative to neutral baseline. The data are thresholded at *p* < 0.05 and FWE corrected at the cluster level. Colourbar indicates the t statistic range

Whole-brain analysis of the PET data revealed higher *BP*_ND_ in the music condition relative to the control condition in several brain regions, including ventral striatum, amygdala, parahippocampal gyrus, thalamus, brainstem, orbitofrontal cortex, and temporal pole (Fig. 3, Table S1). The opposite contrast did not reveal significant effects.

**Fig. 4:**
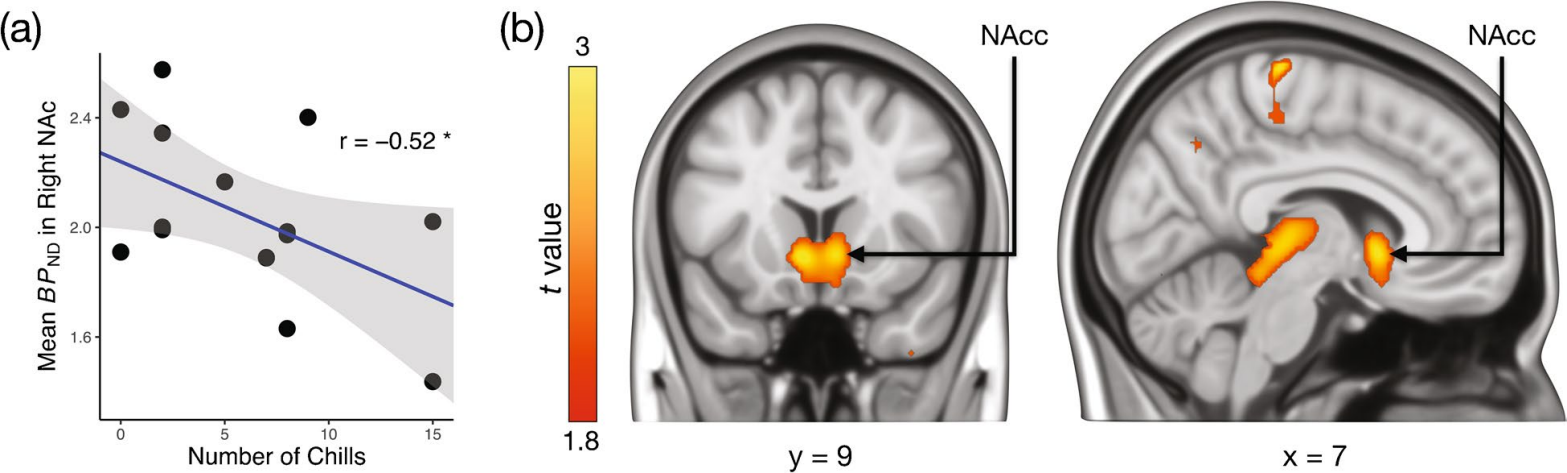
(**a**) Scatterplot illustrating the relationship between the number of chills and mean *BP*_ND_ in right nucleus accumbens (NAcc). (**b**) Voxels showing a negative association between the number chills and [^11^C]carfentanil *BP*_ND_ during the music condition, thresholded at *p* < 0.05 (uncorrected). Colourbar indicates the t statistic range

Mean *BP*_ND_ during the music scan in the right nucleus accumbens was negatively associated with the number chills participants experienced during music listening (*r* = −0.52, *p* < 0.05) (Fig. 4a). This negative association between tracer binding in the bilateral NAcc and chills was corroborated by a voxel-wise second-level analysis of the *BP*_ND_ maps from the music scans, using the number of chills as a covariate (Fig. 4b), although the result was significant only without cluster correction. While a few other regions, such as the thalamus, also showed significant voxels under this lenient thresholding in the voxel-wise analysis, we focus on the NAcc due to a strong a priori hypothesis regarding its role in opioid-mediated pleasure.

### fMRI

**Fig. 5:**
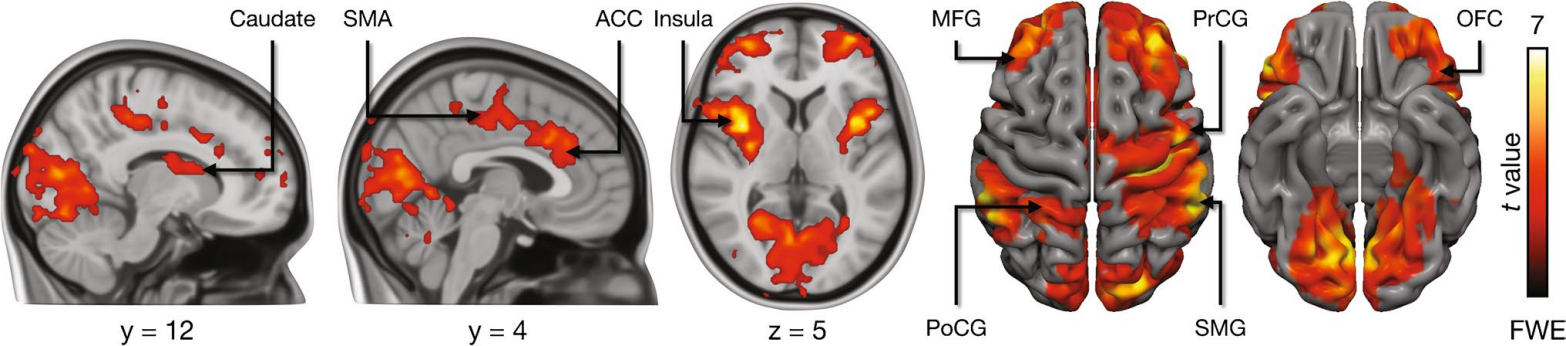
Pleasure-dependent BOLD responses. The data are thresholded at *p* < 0.05 and FWE corrected at cluster level. ACC = Anterior cingulate cortex, MFG = Middle frontal gyrus, PoCG = Postcentral gyrus, PrCG = precentral gyrus, SMA = Supplementary motor area, SMG = supramarginal gyrus, OFC = Orbitofrontal gyrus. Colourbar indicates the t statistic range

Modeling the BOLD data with the parametric dynamic pleasure ratings during the music and control blocks revealed activity in the insula, orbitofrontal cortex (OFC), and ACC, as well as in the middle frontal gyrus and frontal pole. Subcortically, activation was observed particularly in right caudate and bilateral putamen. Activation was also seen in sensorimotor regions in the pre and postcentral gyri, SMA, and supramarginal gyrus as well as in the visual cortex (Fig. 5).

### PET-fMRI fusion analysis

**Fig. 6:**
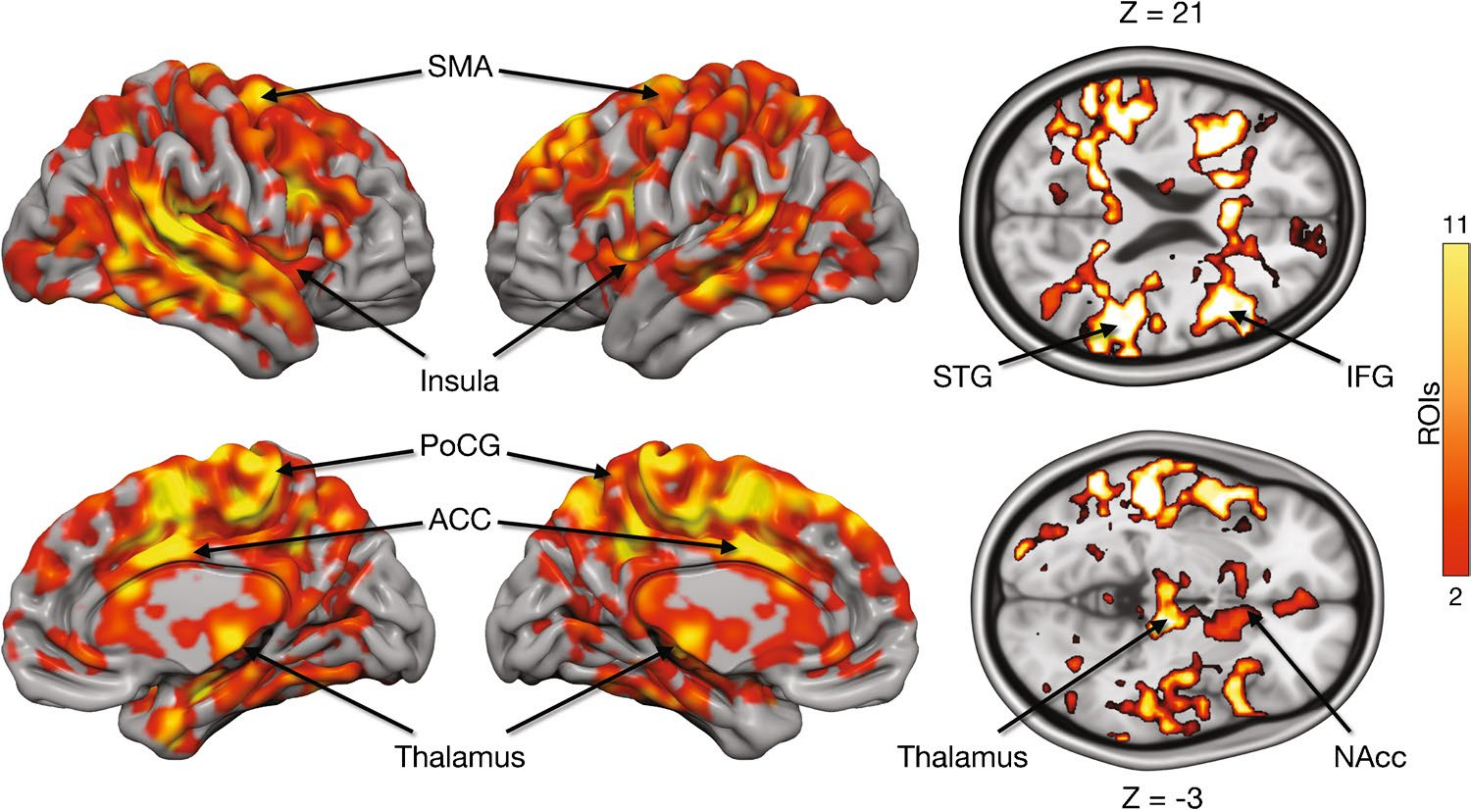
Cumulative maps showing the number of ROIs (out of 17) where *BP*_ND_ was positively correlated with pleasure-dependent BOLD responses. ACC = Anterior cingulate cortex, PoCG = Postcentral gyrus, SMA = Supplementary motor area

Finally, we tested whether regional baseline MOR availability in 17 a priori selected ROIs was associated with the whole brain pleasure-dependent BOLD responses. This analysis revealed significant positive associations with the regional *BP*_ND_ and BOLD responses in the Insula, ACC, SMA, post central gyrus, and middle and superior temporal gyrus, ventral striatum, and thalamus (Fig. 6). No significant negative associations between MOR tone and haemodynamic responses were found.

## Discussion

Our main finding was that pleasurable music modulated MOR system activity in several cortical and subcortical regions and that pleasurable chills during music listening were associated with* BP*_N__D_ in the nucleus accumbens, linking striatal MOR release to subjective experience. The fMRI results revealed that acutely music-induced pleasure was associated with activity in regions implicated in interoception, emotion, and reward. Finally, the PET-fMRI fusion analysis demonstrated that higher baseline MOR availability was associated with stronger pleasure-dependent BOLD responses particularly in the reward circuit as well as in motor regions. Altogether, our results represent the first in vivo neuroimaging evidence supporting the mediating role of the MOR system in the experience of music-induced pleasure and underline how interindividual variability in the MOR system is associated with individual differences in the tendency for reward responses to music.

### Music modulates MOR system function

The PET data revealed that pleasurable music modulated opioidergic activity in central nodes of the reward circuits such as the ventral striatum and OFC containing”hedonic hotspots” regulated by opioids [9]. While prior [^11^C]carfentanil PET studies on reward processing have mainly focused on biologically salient primary rewards such as feeding [32], sex [14], and sociability [15], our results indicate that aesthetic rewards, such as music, that are strongly influenced by cultural learning, also modulate MOR system activity. This suggests that MORs mediate pleasure across domains, driving humans to pursue primary and non-primary rewards and make choices in the face of conflicting options [33]. Due to the well-established role of endogenous opioids in affiliative behavior and social rewards, the MOR system has been hypothesized to contribute particularly to the prosocial effects of musical activities involving synchronized movements in groups, such as joint music-making and dancing [2, 20]. Our results, however, indicate that even solitary listening to pleasurable music without overt movement can modulate MOR system activity.

We observed higher *BP*_ND_ in the music condition compared to the control condition. A decrease in *BP*_ND_ is typically interpreted as evidence for heightened endogenous neurotransmitter release, in line with competition between the radiotracer and synaptic neurotransmitters [34, 35], while an elevation in [^11^C]carfentanil binding may signify MOR "deactivation", a reduction in synaptic endogenous opioids [25]. However, *BP*_ND_ encompasses both receptor density and affinity, and augmented radioligand binding may indicate an increase in the available receptors or enhanced binding affinity. While prior studies indicate that pleasurable stimuli often decreases [^11^C]carfentanil *BP*_ND_, pleasurable touch, laughter, and social acceptance [15, 25, 36] have also been reported to heighten regional MOR availability. The current design does not allow us to disentangle whether the observed effects are due to increased or decreased opioid tone.

However, we found that the number of music-induced chills was negatively correlated with [^11^C]carfentanil binding in the nucleus accumbens. As chills are widely recognized as a proxy for intense music-induced pleasure, this finding indicates that such pleasurable experiences are associated with increased endogenous opioid release in the NAcc, which competes with carfentanil for receptor binding [34]. This result aligns with animal studies demonstrating the central role of opioid release in the NAcc in mediating pleasure and parallels evidence of striatal dopamine release during music-induced pleasure [6]. The increased [^11^C]carfentanil binding in the music scan relative to the baseline scan, may reflect a more general effect, such as heightened arousal, rather than pleasure specifically. In contrast, the observed opioid release in the NAcc appears to be more directly tied to the pleasurable aspects of the music experience itself.

While endogenous opioids have often been hypothesized to mediate musical hedonia [5, 17, 37], an alternative proposal is that the dopamine system constitutes the main neurochemical pathway for music-induced pleasure [28]. This assertion is supported by a PET study demonstrating heightened dopamine release during music-induced chills [6] and pharmacological data showing that dopamine receptor blockade diminishes music-induced pleasure and dopamine agonist administration had the opposite effect [38]. Animal studies have, however, demonstrated that injecting opioid agonists into the striatum increases liking reactions, whereas dopamine antagonist injections in these sites or chemical (6-OHDA) lesions of the striatal dopaminergic neurons do not reduce liking responses [9, 39]. Moreover, one human study has reported opioid antagonist-dependent weakening of pleasure induced by music [26], yet this outcome has not been replicated in subsequent research [27, 28]. The dopamine and opioid systems interact at the molecular level [40]. For instance, opioid release in the ventral tegmental area (VTA) has been shown to modulate dopamine release in the nucleus accumbens (NAc), providing a potential pathway through which both systems could contribute to music-induced pleasure [41]. It is thus likely that the interplay the opioid and dopamine system, and possible interactions with other systems such as oxytocin [42], plays a role in shaping the experience of music-induced pleasure and arousal [37].

### MOR tone is associated with haemodynamic responses to pleasurable music

Our fMRI results revealed that activity in the orbitofrontal cortex (OFC) and dorsal striatum correlated with the subjective experience of music-induced pleasure. Prior research shows that OFC activity tracks subjective pleasure from rewards like food and sexual cues [9]. The dorsal striatum, the caudate and putamen are consistently activated by rewards, including liked music [30, 43, 44].

Pleasure-dependent activation was also observed in the ACC and insula [44, 45], linked to visceral signal processing and the physiological arousal accompanying music-induced pleasure [46, 47]. Pleasurable music elicited increased heart rate and stronger pupillary responses, indicating heightened autonomic activity, consistent with prior studies showing that music-induced pleasure coincides with emotional arousal [48]. Insula activation, common to music and food rewards [4], may reflect its role in emotional and bodily responses central to music. Activation in the right pre/postcentral gyri and supramarginal gyrus indicates somatomotor system involvement, mirroring bodily sensations or movement simulations triggered by liked music [49, 50]. The pleasure-dependent BOLD responses co-localized with music-induced *BP*_ND_ changes in the ACC, and frontal pole, among other regions, suggesting that increased blood flow during music-induced pleasure may partly reflect the MOR system's metabolic demands.

Baseline MOR availability was associated with hemodynamic pleasure-dependent responses: Participants with a higher concentration of MORs exhibited stronger haemodynamic pleasure responses, particularly in the ACC, insula, and auditory cortex as well as in the NAc. Thus, our results indicate that individual variation in MOR tone influences pleasure responses in regions associated with bodily, auditory, and reward processing during pleasurable music listening, which may explain individual differences in subjective music-induced emotional experience. It is noteworthy, however, that prior studies have shown that baseline MOR availability is associated with functional BOLD responses to both positive, reward-related stimuli, such as food pictures and laughter sounds, as well as negative stimuli, like violent videos [16, 31, 51]. MOR availability has also been associated with haemodynamics responses reflecting emotional arousal irrespective of valence. Together, these findings suggest the endogenous opioid system has a highly general role in modulating the processing of a wide range of emotionally evocative events irrespective of valence and biological saliency.

### Limitations

Due to the complexity of PET neuroreceptor imaging, our sample size was relatively small, which may have compromised the ability to detect small effects. However, the PET results were robust, consistent across participants, and localized in all the expected regions, suggesting that the statistical power allowed us to detect biologically meaningful effect sizes. For reasons outlined in the supplementary methods, we only included female participants, which may limit the generalizability of our results to males.

## Conclusions

We conclude that the endogenous opioid system plays a modulatory role in music-induced pleasure. This was evident through i) observed changes in endogenous MOR tone during pleasurable music listening in the PET experiment, ii) correlation between MOR availability in the nucleus accumbens and music induced chills, and iii) the ability of MOR tone to modulate pleasure-dependent hemodynamic responses in fMRI. These results underscore the involvement of MORs not only in primary reward processing but also in mediating pleasure responses to abstract, aesthetic rewards. Clinical studies should further explore whether MOR signaling mediates the analgesic, emotional, and cognitive effects of music-based interventions for pain and neuropsychiatric disorders.

## Supplementary Information

Below is the link to the electronic supplementary material.

Supplementary file1 (DOCX 126 KB)

## Data Availability

The datasets generated during and/or analysed during the current study are available from the corresponding author on reasonable request.
