# Supplementary material for: Pleasurable music activates cerebral µ-opioid receptors: a combined PET-fMRI study
Source: Eur J Nucl Med Mol Imaging. 2025 Apr 4;52(10):3540–9. doi: 10.1007/s00259-025-07232-z (PMC12316753; doi:10.1007/s00259-025-07232-z)
Supplement: Supplementary file 1 — Supplementary file1 (DOCX 126 KB) [file 259_2025_7232_MOESM1_ESM.docx]

**Supplementary Materials for**

**Pleasurable music activates cerebral µ-opioid receptors: A combined PET-fMRI study.**

Vesa Putkinen, Kerttu Seppälä, Harri Harju, Jussi Hirvonen, Henry K., Karlsson, and Lauri Nummenmaa

**Supplementary Methods**

**Participant recruitment and screening**

The participants were recruited through university email lists. To ensure that the participants would enjoy listening to music, potential participants filled in the Emotional Evocation, Mood Regulation, and Social Reward facets of the Barcelona Music Reward Questionnaire (BMRQ)^1^, and those who received at least 80% of the possible maximum score were invited to participate. Due to the extensive screening already required for participation in the PET study, only some facets of the BMRQ were used. These were selected specifically for their direct relevance to music-induced emotions and reward, rather than to music-induced movement or music-seeking. Only women were studied to maximize statistical power due to sex-dependent variability in the spatial distribution of MORs^2^. Additionally, women report stronger emotional responses^3^, thus female-only sample was assumed to maximize the effects in the complex PET studies. The study physician screened the participants for eligibility, and a psychologist screened them for psychiatric disorders with the M.I.N.I 6.0 interview^4^. The exclusion criteria included a history of neurological or psychiatric disorders, alcohol and substance abuse, current use of medication affecting the central nervous system, and the standard MRI exclusion criteria. Structural brain abnormalities that are clinically relevant or could bias the analyses were excluded by a consultant neuroradiologist.

**PET image processing**

PET data were preprocessed with the Magia^5^ toolbox (<https://github.com/tkkarjal/magia>) running on MATLAB (The MathWorks, Inc., Natick, MA, USA). PET images were first motion-corrected and coregistered to T1-weighted (T1w) MR images, after which T1w image was processed with Freesurfer for anatomical parcellation. [^11^C]carfentanil uptake was quantified as the binding potential (*BP*_ND_) relative to non-displaceable binding, estimated with the simplified reference tissue model (SRTM) at voxel-level by using the occipital cortex as the reference region. *BP*_ND_ images were spatially normalized to MNI152-space and smoothed using a Gaussian kernel (FWHM = 6 mm).

**MRI data acquisition and preprocessing**

The MRI data were acquired using a 3T MRI system with SuperG gradient technology (SIGNA, Premier, GE Healthcare, Waukesha, WI, USA) with the 48-channel head coil. High-resolution structural images were obtained with a T1-weighted (T1w) MPRAGE sequence (1 mm^3^ resolution, TR 7.3 ms, TE 3.0 ms, flip angle 8◦, 256 mm FOV, 256 × 256 reconstruction matrix). 556 functional volumes (24 min) were acquired with a T2∗-weighted echo-planar imaging sequence sensitive to the blood-oxygen-level-dependent (BOLD) signal contrast (TR 2600 ms, TE 30 ms, 75◦ flip angle, 240 mm FOV, 80 × 80 reconstruction matrix, 3.0 mm slice thickness, 45 interleaved axial slices acquired in descending order without gaps).

Functional imaging data were preprocessed with FMRIPREP. During preprocessing, each T1w volume was corrected for intensity non-uniformity using N4BiasFieldCorrection (v2.1.0) and skull-stripped using antsBrainExtraction.sh (v2.1.0) using the OASIS template. Brain surfaces were reconstructed using recon-all from FreeSurfer (v6.0.1), and the brain mask estimated previously was refined with a custom variation of the method to reconcile ANTs-derived and FreeSurfer-derived segmentations of the cortical grey matter of Mindboggle. Spatial normalization to the ICBM 152 Nonlinear Asymmetrical template version 2009c was performed through nonlinear registration with the antsRegistration (ANTs v2.1.0), using brain-extracted versions of both T1w volume and template. Brain tissue segmentation of cerebrospinal fluid, white matter and grey matter was performed on the brain-extracted T1w image using FAST (FSL v5.0.9).

**Heart rate and pupil size measurements**

We assessed autonomic nervous system activation by comparing mean heart rate during the music and baseline PET scans. Heart rate was measured using a Polar M430 GPS running watch, and a Polar H10 heart rate sensor.

In the eye-tracking experiment, we measured pupil size with Eye Link II system with 250 Hz sampling rate and spatial accuracy better than 0.5 degrees. The recording was conducted in a dimly lit room. The participants were seated with their chin on a chin rest. The eye tracker was calibrated and validated using standard 9-point calibration. The participants listened to 10 60-sec excerpts of their self-chosen pleasurable music and six 45-sec control stimuli used in the fMRI experiment (see above). A control stimulus was presented after 2-3 music excerpts. Each trial began with a drift correction and detrending. Participants were instructed to keep their eyes fixated at a cross shown at the center of the screen while listening. Gaze position and pupil size were measured throughout the trial, after which the subject reported using a keyboard their experience of liking, calmness, and feeling energized on a scale from 1 to 4 (1 = very weak, 4 = very strong) (**Figure S2**). The eye tracker was recalibrated at the middle of the experiment. Subject-wise pupil size time series were cleaned from blinks using in-house code based on PhysioData Toolbox^6^, baseline corrected (0-20 ms), and mean pupil sizes between 2 and 10 seconds was compared across the music and control trials.

**Figure S1.** The genres for the music selected by the subjects.

**Figure S2.** Mean ratings for liking, energization and calmness for the musical excerpts and control stimuli in the eye-tracking experiment.

**Table S1.** Mean t values and effects sizes for anatomical regions-of-interest (ROIs) defined using the Harvard-Oxford atlas.

| ROI | Mean *t* Value | *r* | Cohen's D |
| --- | --- | --- | --- |
| Music > Baseline MOR binding | | | |
| Amygdala | 3.473 | - | 0.897 |
| OFC | 3.031 | - | 0.783 |
| NAcc | 2.694 | - | 0.696 |
| Thalamus | 2.626 | - | 0.678 |
| Chills – BP_ND_ correlation | | | |
| NAcc (right) | - | -.52 | 1.218 |
| NAcc (left) | - | -.49 | 1.124 |

**References**

1. Mas-Herrero E, Marco-Pallares J, Lorenzo-Seva U, Zatorre RJ, Rodriguez-Fornells A. Individual Differences in Music Reward Experiences. *Music Percept Interdiscip J*. 2013;31(2):118-138. doi:10.1525/mp.2013.31.2.118

2. Kantonen T, Karjalainen T, Isojärvi J, et al. Interindividual variability and lateralization of μ-opioid receptors in the human brain. *NeuroImage*. 2020;217:116922. doi:10.1016/j.neuroimage.2020.116922

3. Lench HC, Flores SA, Bench SW. Discrete emotions predict changes in cognition, judgment, experience, behavior, and physiology: a meta-analysis of experimental emotion elicitations. *Psychol Bull*. 2011;137(5):834.

4. Sheehan DV, Lecrubier Y, Sheehan KH, et al. The Mini-International Neuropsychiatric Interview (MINI): the development and validation of a structured diagnostic psychiatric interview for DSM-IV and ICD-10. *J Clin Psychiatry*. 1998;59(20):22-33.

5. Karjalainen T, Tuisku J, Santavirta S, et al. Magia: Robust Automated Image Processing and Kinetic Modeling Toolbox for PET Neuroinformatics. *Front Neuroinformatics*. 2020;14. Accessed April 12, 2022. https://www.frontiersin.org/article/10.3389/fninf.2020.00003

6. Kret ME, Sjak-Shie EE. Preprocessing pupil size data: Guidelines and code. *Behav Res Methods*. 2019;51(3):1336-1342. doi:10.3758/s13428-018-1075-y
